# Academic Primer Series: Five Key Papers about Team Collaboration Relevant to Emergency Medicine

**DOI:** 10.5811/westjem.2016.11.31212

**Published:** 2017-01-20

**Authors:** Michael Gottlieb, Catherine Grossman, Emily Rose, William Sanderson, Felix Ankel, Anand Swaminathan, Teresa M. Chan

**Affiliations:** * Rush University Medical Center, Department of Emergency Medicine, Chicago, Illinois; † Virginia Commonwealth University Health Systems, Department of Internal Medicine, Richmond, Virginia; ‡ University of Southern California Keck School of Medicine and Medical Center, Department of Emergency Medicine, Los Angeles, California; § University of Kentucky, Department of Emergency Medicine, Lexington, Kentucky; ¶ HealthPartners Institute, Health Professions Education, Bloomington, Minnesota; || University of Minnesota Medical School, Department of Emergency Medicine, Minneapolis, Minnesota; # New York University School of Medicine, Department of Emergency Medicine, New York, New York; ** McMaster University, Department of Medicine, Division of Emergency Medicine, Hamilton, Ontario, Canada

## Abstract

**Introduction:**

Team collaboration is an essential for success both within academics and the clinical environment. Often, team collaboration is not explicitly taught during medical school or even residency, and must be learned during one’s early career. In this article, we aim to summarize five key papers about team collaboration for early career clinician educators.

**Methods:**

We conducted a consensus-building process among the writing team to generate a list of key papers that describe the importance or significance of team collaboration, seeking input from social media sources. The authors then used a three-round voting methodology akin to a Delphi study to determine the most important papers from the initially generated list.

**Results:**

The five most important papers on the topic of team collaboration, as determined by this mixed group of junior faculty members and faculty developers, are presented in this paper. For each included publication, a summary was provided along with its relevance to junior faculty members and faculty developers.

**Conclusion:**

Five key papers about team collaboration are presented in this publication. These papers provide a foundational background to help junior faculty members with collaborating in teams both clinically and academically. This list may also inform senior faculty and faculty developers about the needs of junior faculty members.

## INTRODUCTION

Team collaboration is essential to practicing emergency medicine (EM). It is necessary in the clinical environment when managing sick patients, as well as in the academic environment when running projects or creating scholarship.^1^,^2^ Unfortunately, team collaboration is infrequently taught in medical school or residency, leaving the early clinical educator with few resources from which to develop and enhance these skills.^3^ This is one of several needs identified by early clinical educators.^4^

The Academic Life in Emergency Medicine (ALiEM) Faculty Incubator was created in 2016 in an effort to address some of the issues that junior faculty members often face. During our one-year experience, we created modules in which we described and discussed key literature relevant to junior clinician educators who are embarking in their careers within academic medicine. This particular paper is a synthetic, narrative review that highlights some of the most important literature on the topic of team collaboration, which was the second topic covered in our discovery-based curriculum. The objective of this paper was to summarize five key papers about team collaboration to both inform on key concepts and identify techniques for improving teamwork.

## METHODS

In the second month of the ALiEM Faculty Incubator, the topic of team collaboration was discussed. The ALiEM Faculty Incubator consists of 30 junior faculty members and eight facilitators (faculty mentors and administrators) who exist via a closed, mixed-media, social media platform (Slack.com, San Francisco, CA). The platform allows for text-based communication, augmented by file-sharing and embedded website links. The discussion that occurred involved an international group of clinician educators spanning three countries (United States, Canada, and Chile) and multiple time zones.

For this publication, we used a method similar to our previous Academic Primer series paper.^5^ We monitored the proceedings of the ALiEM Faculty Incubator from April 1–30, 2016, during which time all members participated asynchronously online in various discussions around the topic of team collaboration. During this month, 2,513 messages were posted. There were no in-person meetings.

**Figure 1:**
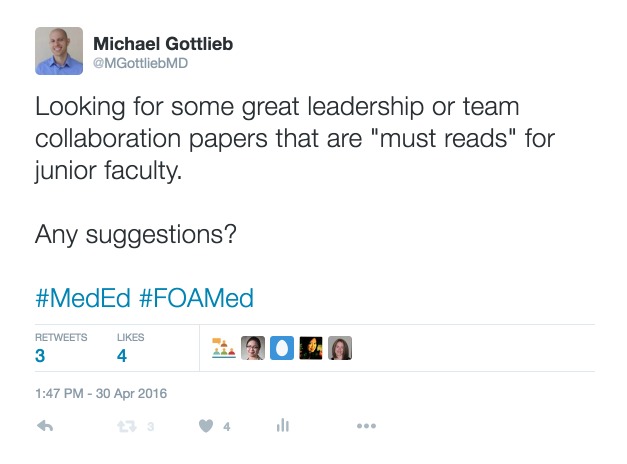
Example of an exemplar Tweet.

We allowed the discussion to unfold and gathered the titles of the papers that were cited, shared, suggested, or discussed within the online discussion forum. This list was then augmented with a general call for suggestions via multiple authors using Twitter to optimize our literature list. We tweeted and retweeted multiple requests to have participants of the #FOAMed and #MedEd online communities provide suggestions for important papers on the topic of team collaboration with specific relevance to junior EM educators. Figure 1 shows an exemplar request tweet.

The authorship team was composed of seven members, consisting of four novices (i.e. junior faculty members) and three experts in the field (i.e., experienced clinician educators, all of whom have published >10 peer-reviewed publications). The expert group was pre-selected based upon significant expertise in the field, while the junior group was hand-selected by the topic experts for that month based upon significant contributions and interest related to the monthly topic. The authors had no major conflicts of interest to disclose. One of the authorship team members (FA) was a co-author of one of the selected papers, but exclusion of his votes did not significantly affect the ranking.

Once the list of the most important papers about teamwork was created, our authorship team conducted a three-round voting procedure inspired by the Delphi methodology. During the third round, there was a tie for the fifth paper, so a fourth round of voting was held with a clear majority favoring the fifth article listed below. The other article was included as an honorable mention. We have not described our method as a pure Delphi methodology since our authorship panel comprises both novices and experts in the field. We intentionally wished to have a mixed group of stakeholders select these articles (i.e., both novices and experts) in order to find articles that would both meet the approval of experienced clinician educators and resonate with junior faculty members entering the field of academic medicine.

## RESULTS

Our initial review of the ALiEM Faculty Incubator discussions yielded a total of 12 articles mentioned by the mentors and the junior faculty participants. Social media calls over one week (April 30, 2016 – May 6, 2016) yielded five additional suggested articles, which led to a total of 17 articles for evaluation by our team. The four-round voting procedure allowed our team to generate a rank-order listing of all these papers in order of relevance from the most important to the least important. The citations and our ratings of these 17 papers are listed in Table.

## DISCUSSION

The following are a summary of the top five papers accompanied with commentaries about their relevance to both junior faculty members and considerations for faculty developers when discussing these works.

### 1. Edmondson AC. Teamwork on the fly. *Harvard Business Review*. 2012 Apr;90(4):72–80.^6^

#### Summary

**Figure 2:**
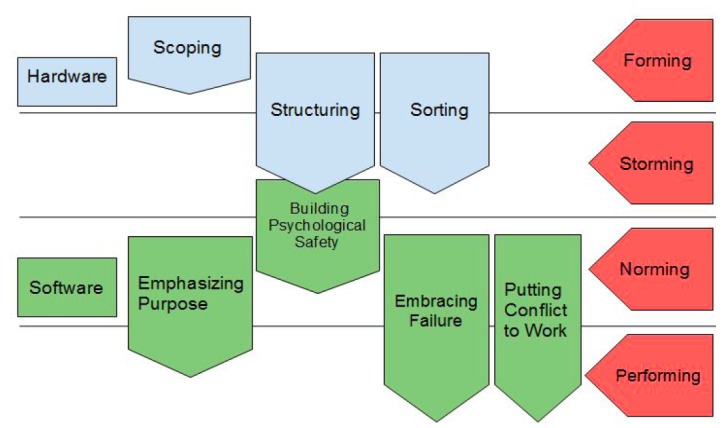
Diagram of the hardware and software model of teaming.

This paper is a distillation of Amy Edmondson’s study of teamwork that describes the concept and skills required to master “teaming.” Teaming addresses the challenges and skills needed of working in ad hoc teams, often dealing with multiple differences among team members: geographical hurdles, levels of expertise, varied disciplines, and possibly cultural norms. This paper then defines principles of teaming using a hardware and software analogy. The “hardware” (required overall project management) is broken down into leadership scoping out the challenge, implementation of light scaffolding to help the team function effectively, and sorting of tasks by priority for execution. “Software” (the team leadership and team followership behaviors that allow teams to be successful) includes emphasizing purpose (shared goal), creating an environment of psychological safety for team members, embracing failure, and putting conflict to work. Furthermore, successful individual-based teaming behaviors include speaking up, listening intently, integrating information/ideas, experimenting, and reflecting. Undercurrents of Tuckman’s stages of group development are woven throughout the article; for example, group “forming” and parts of “storming” would fall under hardware, and the software skills would cover group “storming,” “norming” and “performing” (Figure 2).^7^

#### Relevance to Junior Faculty Members

Junior faculty are often pulled in many directions to both provide service to their institution and engage in academic endeavors towards promotion. Most of this work is done in ad hoc teams. Hospital-wide initiatives, working on projects within single departments, and networking with colleagues at different institutions would all qualify as needing teaming skills. This paper provides specific examples for behavior and structure that allows for work in ad hoc teams, and clear definitions of the elements of teaming. Although the longitudinal example played out in this article centers around an architectural and engineering problem, the concepts are generalizable to “teaming” within teams that physicians find themselves on - direct patient care and non-direct patient care based. Mastering the principles of teaming and reflecting on teaming function and dysfunction could help junior faculty use their time wisely to be more productive on ad hoc teams and possibly create more functional ad hoc teams.

#### Considerations for Faculty Developers

Healthcare is moving from a culture focused on individual exceptionalism to team-based care designed to deliver high-value care. Academic promotion and tenure committees are slowly following suit and are starting to recognize team-based scholarship. The faculty of tomorrow need to achieve competence in basic teaming behaviors to succeed in this environment. Faculty developers can help embed the basic teaming behaviors in departmental culture by valuing and celebrating teaming behavior, both clinically and academically, and creating faculty development programs focusing on teaming behavior. Faculty developers can also mentor junior faculty to navigate the tension between individual exceptionalism valued by traditional medical school promotion and tenure committees and teaming behaviors that are valued by health systems that ultimately hire many of the residency graduates of academic programs.

### 2. Farley H, Casaletto J, Ankel F, et al. An assessment of the faculty development needs of junior clinical faculty in emergency medicine. *Acad Emerg Med*. 2008 Jul;15(7):664–8.^8^

#### Summary

Junior clinical faculty in EM were surveyed to identify and rank importance of self-perceived career development needs. Identified needs included bedside teaching, lecture development, business skill, managerial skills, educational research, mentorship and career counseling, interpersonal skills, leadership skills, scholarly writing skills, physician wellness, and knowledge of the faculty development process. The authors also searched for available resources to address the identified career development needs. The majority of the needs identified had available educational opportunities through the American Academy of Emergency Medicine (AAEM), American College of Emergency Physicians (ACEP), Council for Emergency Medicine Residency Directors (CORD), and Society for Academic Emergency Medicine (SAEM) via national conferences and web-based educational resources. Physician wellness and mentoring were two career development areas identified as scarcely available resources during this review.

#### Relevance to Junior Faculty Members

The path to a productive academic career is often mysterious to a junior faculty member. Navigating the field and creating one’s niche can be challenging, particularly in the early career when the novice may even be uncertain regarding his/her area of interest. Structure to the course of faculty development helps improve the efficiency of the development process. Junior faculty need assistance in development of both short-term and long-term goals. Academic advancement is facilitated by direction and monitored by goal-oriented progress. Specific faculty development resources aid in this process. Unfortunately, many faculty development resources are underutilized.

This article emphasizes that many resources are currently available to junior faculty. Many of them are unaware of several important resources currently in place to facilitate personal development. However, resources alone cannot substitute for mentorship and concrete faculty development goals. Invested mentors are an integral part to facilitate junior faculty development. Mentors offer direction, can help individualize a career roadmap and often provide opportunities to get one’s “foot in the door.”

#### Considerations for faculty developers

Faculty development and physician wellness are interrelated and integral to career satisfaction, vitality, and longevity. Academic physicians are often more productive if they are engaged, challenged and continually growing in knowledge and skills. As the authors state, “ongoing professional development is the mainstay of a successful and satisfying career in academic medicine.”

This article emphasizes that there is either a lack of awareness of available faculty development resources and/or the resources do not meet the intended needs of junior faculty. Faculty developers should be inspired to innovate and invigorate current resources and also fill in the gap, particularly in the identified areas of mentorship and wellness.

### 3. Sargeant J, Loney E, Murphy G. Effective interprofessional teams: “contact is not enough” to build a team. *J Contin Educ Health Prof*. 2008 Fall;28(4):228–34.^9^

#### Summary

This is a qualitative analysis paper focused on identifying themes emerging from dedicated interprofessional focus groups. Assessments of interprofessional educational interventions and collaborations have demonstrated the value of optimizing team dynamics in improving learner knowledge and educational outcomes. To this end, this paper identifies five key characteristics that emerged among effective interprofessional healthcare teams. These include the following:

1. Understanding and respecting team members’ roles
2. Appreciating that teams require more work than expected
3. Understanding the healthcare system or systems in which the team members work
4. Having the practical “know-how” to identify the correct team member for each task within the system
5. Having the ability to use appropriate communication skills to achieve the ends noted above; effective communication ties together and supports the foundation upon which the other characteristics can flourish.

#### Relevance to Junior Faculty Members

While this paper focuses primarily on the primary care physician, the junior faculty member within an emergency department is uniquely positioned to effect change at an institutional level; this necessarily involves multiple disciplines. Oftentimes, the traditional role of physician as leader and superior doesn’t lend itself well to a sense of psychological safety within the group; perceptions of inequality within a team can present a challenge and serve as an impediment to teamwork in groups with non-physician members. An awareness of this is critical to the junior faculty member’s effective integration into interprofessional teams.

#### Considerations for Faculty Developers

This paper discusses several themes that emerged from focus groups tasked with identifying key commonalities experienced when group members recalled examples of effective teaming. While the concepts discussed are not particularly novel, the paper does identify an underserved area within medical education and faculty development: development of teamwork skills in an interprofessional environment. Very little training is dedicated to this area of professional development in traditional medical education; for junior faculty members who lack these skills or who have not undergone formal training in this area, the faculty developer is presented with an opportunity for early intervention.

### 4. Kotter JP. Leading change: Why transformation efforts fail. *Harvard Business Review*. 2007 Jan;85(1):2–12.^10^

#### Summary

This was a narrative review paper from the business literature, which describes eight steps for leading transformative changes. The author discusses the following eight steps: 1) establishing a sense of urgency; 2) forming a powerful guiding coalition; 3) creating a vision; 4) communicating the vision; 5) empowering others to act on the vision; 6) planning for and creating short-term wins; 7) consolidating improvements and producing still more change; and 8) institutionalizing new approaches. The author also emphasizes the importance of sufficient time spent on planning and ensuring sufficient buy-in when enacting large changes.

#### Relevance to Junior Faculty Members

Junior faculty members are often in a great position to identify potential changes within an institution. As new faculty, they can provide an external view to existing curriculum or faculty development approaches, bring new knowledge and approaches from outside programs, and are closer to residents, allowing for an improved ability to identify with current resident and student needs. However, junior faculty also face the challenge of being new to the hospital without the experience or social capital of more senior faculty. This can make change difficult to instill.

This article discusses eight techniques, that have been successful for enacting change in similar scenarios in the business world. The author highlights the need for a cohort of project champions at various levels within the institution, as well as the importance of communicating and maintaining a consistent vision throughout the different academic ranks.

For example, if a few of the faculty consistently disparage a new change to the conference schedule, the negativity can rapidly spread to residents and other faculty, leading to conflicting messages and reduced overall buy-in. It is important to address these issues actively and quickly to ensure successful change. Additionally, one should build in small victories throughout any major change. Because significant changes require time to produce results, plan for and emphasize smaller wins throughout the process, such as resident or patient satisfaction surveys, pre- and post-test surveys, or congratulatory emails.

#### Considerations for Faculty Developers

This is a classic leadership and team management article that is a must-read for all aspiring leaders. The trick to teaching or coaching with this article is to use your local environment and local innovations to help bring these concepts to life for your junior faculty members. Using Kotter’s eight steps as a conceptual framework for analyzing or prompting change with an upcoming innovation or educational program is a great way to help a junior faculty member think through their change process. Each step has actionable items that can be considered, and using a worksheet based on this model (see Appendix) can help your junior colleagues think through their own project and how they can anticipate their next steps for making changes based on their work.

### 5. Fernandez R, Kozlowski SW, Shapiro MJ, et al. Toward a definition of teamwork in emergency medicine. *Acad Emerg Med*. 2008 Nov;15(11):1104–12.^11^

#### Summary

This was a good overview article derived from the *Academic Emergency Medicine* Consensus Conference with a goal of describing and defining teamwork within EM. The authors discuss teamwork using the I-P-O model (input-process-output), wherein input refers to abilities and existing experience of team members, process represents the behaviors and actions, and outputs consist of performance and team satisfaction. Within this model they discuss a variety of necessary components, including assessment of available resources, clearly identifying and assigning roles, contingency planning, team adaptability, monitoring progress, keeping track of resources, workload distribution, and coordination of efforts. Additional supporting components include the importance of leadership, team awareness, and using closed-loop communication.

#### Relevance to Junior Faculty Members

Teamwork is an essential skill for junior faculty. While this article emphasized teamwork within the context of a medical resuscitation, it can be applied to multiple different types of teams. The importance of planning and strong communication both during resuscitations and when creating academic projects cannot be overemphasized. Performing sufficient needs assessments and discussing available resources (e.g. financial support, protected time, and support staff) can lead to much more successful and rewarding projects. Additionally, aspects such as team awareness and workload distribution can be valuable to ensure projects are continuously moving forward, especially in light of the busy and variable schedules of emergency physicians.

#### Considerations for Faculty Developers

This is a very good overview article that situates teamwork in EM. It focuses heavily on a single team-effectiveness model (the I-P-O model) but nicely guides the reader through the conceptual framework and explains it using a common experience that is likely shared among most junior EM faculty members (e.g., a critical care case that starts with pre-hospital team handover, includes an intubation, and the transition of the case to elsewhere in the hospital).

The challenge of using this article for faculty development will be to then show how this model might be useful in other teaming situations (e.g., How might we apply the I-P-O model to a good research team? What about a curriculum team?). It is clinically oriented, and much thought and preparation on the part of the faculty developer is needed to transition the use of this article beyond the simulation room or resuscitation bay. There is some jargon in this article, although the authors explain each concept throughout the article.

### Honorable Mention: Lerner S, Magrane D, Friedman E. Teaching teamwork in medical education. *Mt Sinai J Med*. 2009 Aug;76(4):318–29.^12^

This article is a fantastic review of teamwork training in medical education. It is a must read for those who want a solid foundation in the history and impetus for why we should be incorporating teamwork teaching in health professionals’ education. It provides a more general overview of opportunities for team teaching across the classroom to clinical environments, covering topics including team-based learning (TBL),^12^ team building exercises and team skills training, and the TeamSTEPPS training tool.^13^ For junior faculty members, this paper provides a solid literature review to catch one up on literature around teamwork in medical education. For faculty developers, this paper may be a very good core article to provide as pre-reading before discussing topics around clinical team leadership and how to teach or coach learners on this.

## LIMITATIONS

The main limitation of our proceedings is that our search strategy was not comprehensive. Although we attempted to gather recommendation from multiple sources (e.g., our expert recommendations, Faculty Incubator discussions, social media), we did not perform an exhaustive, structured literature review. The purpose of this paper, however, was to aggregate several high-yield papers that would serve as a starting point for junior faculty members embarking on their academic careers within EM. We believe that the inclusion of both experts and novices (i.e., end-users) in the selection and evaluation process also allowed for a more inclusive selection. The authors hope that this is a valuable starting point for the reader’s exploration and initial development in this topic.

## CONCLUSION

We have provided a reading list that may be beneficial to improve team collaboration among junior faculty. We hope this paper provides junior clinician educators a broad overview of this important topic, making it more approachable.

## Supplementary Information



## Figures and Tables

**Table:** The complete list of educational scholarship literature collected by the authorship team.

Citation	ROUND 1 initial mean scores (SD) *max score 7*	ROUND 2 % of raters that endorsed this paper	ROUND 3 % of raters that endorsed paper in last round	Top 5 papers
Edmondson AC. Teamwork on the fly. *Harvard Business Review*. 2012 Apr;90(4):72–80.^6^	6.00 (0.82)	100%	100%	1
Farley H, Casaletto J, Ankel F, et al. An assessment of the faculty development needs of junior clinical faculty in emergency medicine. *Acad Emerg Med*. 2008 Jul;15(7):664–8.^8^	5.14 (1.46)	85.70%	100%	2
Sargeant J, Loney E, Murphy G. Effective interprofessional teams: “contact is not enough” to build a team. *J Contin Educ Health Prof*. 2008 Fall;28(4):228–34.^9^	5.14 (0.90)	71.40%	100%	3
Kotter JP. Leading Change: Why Transformation Efforts Fail. *Harvard Business Review*. 2007 Jan;85(1):2–12.^10^	6.14 (0.69)	100%	85.70%	4
Fernandez R, Kozlowski SW, Shapiro MJ, et al. Toward a definition of teamwork in emergency medicine. *Acad Emerg Med*. 2008 Nov;15(11):1104–12.^11^	5.14 (0.69)	57.10%	42.90%	5*
Lerner S, Magrane D, Friedman E. Teaching teamwork in medical education. *Mt Sinai J Med*. 2009 Aug;76(4):318–29.^12^	5.00 (1.30)	57.10%	42.90%	Runner Up
Steinert Y, Naismith L, Mann K. Faculty development initiatives designed to promote leadership in medical education. A BEME systematic review: BEME Guide No. 19. *Med Teach*. 2012;34(6):483–503.^15^	4.86 (1.21)	28.60%	0%	
Fernandez R, Vozenilek JA, Hegarty CB, et al. Developing expert medical teams: toward an evidence-based approach. *Acad Emerg Med*. 2008 Nov;15(11):1025–36.^16^	4.71 (0.95)	42.90%	14.30%	
Bonebright DA. 40 years of storming: a historical review of Tuckman’s model of small group development. *Human Resource Development International*. 2010 Feb;13(1):111–20.^17^	4.57 (0.98)	57.10%	14.30%	
Webb AM, Tsipis NE, McClellan TR, et al. A first step toward understanding best practices in leadership training in undergraduate medical education: a systematic review. *Acad Med*. 2014 Nov;89(11):1563–70.^18^	4.43 (1.27)	28.60%	0%	
Stoller JK. Developing physician-leaders: a call to action. *J Gen Intern Med*. 2009 Jul;24(7):876–8.^19^	4.29 (0.95)	14.30%	0%	
Wolter N, Tarnoff SL, Leckman L. Recruiting and retaining physician leaders. Healthc (Amst). Epub 2015 Oct 20.^20^	4.00 (1.41)	0%	0%	
Hall P, Weaver L. Interdisciplinary education and teamwork: a long and winding road. *Med Educ*. 2001 Sep;35(9):867–75.^21^	3.86 (1.35)	0%	0%	
Sacks L, Margolis R. Physician leadership in organizations undergoing major transformation. Healthc (Amst). Epub 2015 Oct 20.^22^	3.86 (1.35)	0%	0%	
Cochran J, Kaplan GS, Nesse RE. Physician leadership in changing times. Healthc (Amst). 2014 Mar;2(1):19–21.^23^	3.71 (1.11)	0%	0%	
Bisordi J, Abouljoud M. Physician leadership initiatives at small or mid-size organizations. Healthc (Amst). Epub 2015 Oct 20.^24^	3.43 (0.79)	0%	0%	
Bronson D, Ellison E. Crafting successful training programs for physician leaders. Healthc (Amst). Epub 2015 Oct 20.^25^	3.3 (0.95)	0%	0%	

* Due to a tie between two articles during the third round of voting, a fourth round was held between Fernandez et al.^11^ and Lerner et al.^12^ Fernandez et al. was selected as the fifth article for inclusion by a majority of votes.
