# Supplementary material for: Academic Primer Series: Five Key Papers about Team Collaboration Relevant to Emergency Medicine
Source: West J Emerg Med. 2017 Jan 20;18(2):303–10. doi: 10.5811/westjem.2016.11.31212 (PMC5305141; doi:10.5811/westjem.2016.11.31212)
Supplement: Supplementary file 1 [file wjem-18-303-s001.pdf]

Appendix A: A Worksheet for Junior Faculty Members based on Kotter's Eight Steps of Leading Change

**Kotter's Step**	**Questions for the Project Team & Leader**	**Specific Application to Your Project**
1) Establishing a sense of urgency	What are the latest or upcoming problems that are creating a sense of urgency for this change? (e.g. accreditation, resident suggestions, etc.)	
2) Forming a powerful guiding coalition	Who are your key stakeholders? Who are people who have the ability to support or prevent your change from happening?	
3) Creating a vision	What is your vision?<br>What does your innovation or project help you to create?	
4) Communicating the vision	How are you going to communicate your vision?	
5) Empowering others to act on the vision	How are you going to engage your stakeholders in your implementation process to achieve your vision?	
6) Planning for and creating short-term wins	What are some early 'wins' that you can identify for your team?<br><br>What defines early success?	
7) Consolidating improvements and producing still more change	What is your continual quality improvement structure look like?<br><br>What is the project's take on program evaluation?	

8) Institutionalizing new approaches	What are tasks related to your project that you can begin to make ‘routine’ and ‘normal’?<br><br>How can you align your project to current institutional structures?	
